# *Prochlorococcus* Cells Rely on Microbial Interactions Rather than on Chlorotic Resting Stages To Survive Long-Term Nutrient Starvation

**DOI:** 10.1128/mBio.01846-20

**Published:** 2020-08-11

**Authors:** Dalit Roth-Rosenberg, Dikla Aharonovich, Tal Luzzatto-Knaan, Angela Vogts, Luca Zoccarato, Falk Eigemann, Noam Nago, Hans-Peter Grossart, Maren Voss, Daniel Sher

**Affiliations:** a Department of Marine Biology, Leon H. Charney School of Marine Sciences, University of Haifa, Haifa, Israel; b Department of Biological Oceanography, Leibniz-Institute for Baltic Sea Research, Warnemuende, Germany; c Department of Experimental Limnology, Leibniz-Institute of Freshwater Ecology and Inland Fisheries, Stechlin, Germany; d Potsdam University, Institute of Biochemistry and Biology, Potsdam, Germany; University of Georgia

**Keywords:** heterotrophic bacteria, microbial interactions, NanoSIMS, phytoplankton, picocyanobacteria, resting stages

## Abstract

Many microorganisms produce resting cells with very low metabolic activity that allow them to survive phases of prolonged nutrient or energy stress. In cyanobacteria and some eukaryotic phytoplankton, the production of resting stages is accompanied by a loss of photosynthetic pigments, a process termed chlorosis. Here, we show that a chlorosis-like process occurs under multiple stress conditions in axenic laboratory cultures of *Prochlorococcus*, the dominant phytoplankton linage in large regions of the oligotrophic ocean and a global key player in ocean biogeochemical cycles. In *Prochlorococcus* strain MIT9313, chlorotic cells show reduced metabolic activity, measured as C and N uptake by Nanoscale secondary ion mass spectrometry (NanoSIMS). However, unlike many other cyanobacteria, chlorotic *Prochlorococcus* cells are not viable and do not regrow under axenic conditions when transferred to new media. Nevertheless, cocultures with a heterotrophic bacterium, Alteromonas macleodii HOT1A3, allowed *Prochlorococcus* to survive nutrient starvation for months. We propose that reliance on co-occurring heterotrophic bacteria, rather than the ability to survive extended starvation as resting cells, underlies the ecological success of *Prochlorococcus*.

## Abstract (precis)

The ability of microorganisms to withstand long periods of nutrient starvation is key to their survival and success under highly fluctuating conditions that are common in nature. Therefore, one would expect this trait to be prevalent among organisms in the nutrient-poor open ocean. Here, we show that this is not the case for *Prochlorococcus*, a globally abundant and ecologically important marine cyanobacterium. Instead, *Prochlorococcus* relies on co-occurring heterotrophic bacteria to survive extended phases of nutrient and light starvation. Our results highlight the power of microbial interactions to drive major biogeochemical cycles in the ocean and elsewhere with consequences at the global scale.

## INTRODUCTION

Not all microbial cells living in natural environments are equally active. In aquatic environments, up to 90% of the cells do not exhibit measurable metabolic activity (“vitality”), based on dyes (e.g., that assess electron transport) or on uptake assays with labeled substrates (1). Several possible and nonexclusive explanations have been proposed for this heterogeneity. First, observed differences in activity between cells in natural populations may represent inherent differences in activity between genetically different organisms, e.g., due to variations in maximum growth rate or the ability to utilize the specific substrate tested. Second, cells might be at different physiological states, e.g., exponentially growing, starved, or dying, and thus exhibiting different levels of metabolic activity (2, 3). Third, cells show stochastic fluctuations in their activity due to noise in gene expression or regulatory networks (4). Finally, some organisms respond to environmental stress by producing resting stages or spores. Such cells often exhibit very low (or undetectable) metabolic activity and yet are viable, namely, able to return to an active state and reproduce when environmental conditions return to favorable (5). The presence of such resting stages, together with a fluctuating activity at the single-cell level and the genetic variability found within natural populations, is suggested to promote the survival of the population as a whole (2, 6).

Understanding the factors affecting the metabolic activity (vitality) of phytoplankton is of special interest. These microbial primary producers perform about one-half of the photosynthesis on Earth, providing energy through carbon fixation at the base of the aquatic ecosystem (7). As phytoplankton grow, they take up elements such as nitrogen (N) and phosphorus (P) from the environment, potentially leading to low nutrient concentrations that may constrain the growth of both the phytoplankton themselves and co-occurring organisms (8, 9). Phytoplankton viability, including their ability to survive under conditions of nutrient stress, has been extensively studied, especially for organisms that produce massive blooms that emerge and decline rapidly (for reviews, see references 10, 11, and 12). For example, some bloom-forming cyanobacteria, such as *Aphanizomenon* species, produce morphologically distinct spores that show very little photosynthetic activity and yet remain viable in the sediment for long periods of time, providing the inoculum for the next growth season (13). In laboratory cultures of Synechococcus elegantus PCC 7942 and *Synechocystis* PCC 6803, two unicellular freshwater cyanobacteria, nitrogen starvation results in a programmed process where cells enter a resting stage, enabling them to survive prolonged periods of stress (14, 15). As part of this process, cells degrade their photosynthetic apparatus in a controlled manner, resulting in a loss of chlorophyll autofluorescence and culture bleaching (a process termed chlorosis). However, the observation that chlorotic cells are viable resting stages is not universal. Chlorotic cultures of Microcystis aeruginosa PCC 7806 were shown to contain a small population of nonchlorotic cells with high chlorophyll autofluorescence (described throughout this study as “high-fl”). Only these high-fl cells were suggested to revive after the re-addition of a nitrogen source, whereas the low-fl cells are presumably dead (16). Chlorotic cells were also observed in eukaryotic phytoplankton. However, it is not yet clear to what extent such cells remain viable, since it may depend on the specific organism and stress conditions (11, 17, 18).

*Prochlorococcus* is a pico-cyanobacterium that is extremely abundant in the oligotrophic oceans, performing an estimated ∼8.5% of global ocean photosynthesis (19). The carbon fixed by *Prochlorococcus*, which is estimated to produce up to 75% of the daily photosynthetic carbon in the surface layer of the Pacific subtropical gyre (20), can then be utilized by co-occurring heterotrophic bacteria. *Prochlorococcus* cells in the oceans exhibit extremely high genetic diversity (21), and some of this diversity has been linked with their ability to grow under conditions of extreme nutrient limitation (22, 23). It has therefore been suggested that this genetic diversity enables *Prochlorococcus* as a group to thrive across a wide variety of oceanic conditions (24). While the physiological and transcriptional responses of multiple *Prochlorococcus* lineages to short-term nutrient starvation have been extensively studied (22, 25–29), little is known about their ability to survive more than a few days under such conditions. A study on the response of *Prochlorococcus* strains to a different type of stress, extended darkness (i.e., C starvation), has shown that these organisms can survive light starvation only for a limited time (30). In these experiments, low-fl cell populations reminiscent of chlorotic cells in other cyanobacteria appeared after the light-starved cultures were reexposed to light (30). Therefore, phenotypic evidence exists that *Prochlorococcus* can undergo a chlorosis-like process, and yet whether these chlorotic cells are active, and whether they are resting stages that can resume growth when conditions are favorable, is currently unknown. Our experiments were therefore designed to answer the following questions. (i) Do *Prochlorococcus* respond to long-term nutrient starvation by producing chlorotic cells? (ii) If so, are such cells metabolically active (vital), and are they able to reproduce and grow when stress conditions end (viable)? To address these questions, we used fluorescence-activated cell sorting (FACS) to obtain distinct chlorotic subpopulations from axenic and unialgal laboratory cultures of *Prochlorococcus* which were preincubated with isotopically labeled tracers for photosynthesis (H^13^CO_3_) and nutrient uptake (^15^NH_4_^+^), and we visualized their activity using nanoscale secondary ion mass spectrometry (NanoSIMS). This method enabled us to measure photosynthesis and N uptake at single cell resolution by quantifying the change in isotopic ratios (31, 32). Our results show that while *Prochlorococcus* do undergo a chlorosis-like process, with some of the chlorotic cells still photosynthesizing and taking up NH_4_^+^, the chlorotic cells are unable to regrow and thus do not represent resting stages. Instead, coculture with heterotrophic bacteria enables *Prochlorococcus* to survive long-term stress even without producing resting stages.

## RESULTS AND DISCUSSION

### Emergence of chlorotic subpopulations in *Prochlorococcus* cultures.

**FIG 1:**
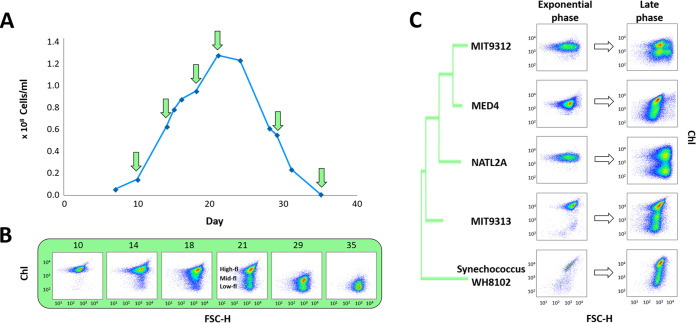
Emergence of chlorotic subpopulations in *Prochlorococcus* batch cultures as measured by flow cytometry. (A) Representative growth curve of an axenic culture of MIT9313, grown in Pro99. The arrows mark the days shown in panel B. (B) Flow cytometry scattergrams at the marked time points from the MIT9313 culture. The *x* axis is the forward scatter (FSC, a proxy for cell size), and the *y* axis is the chlorophyll autofluorescence of the cells (PerCP). The emergence of chlorotic subpopulation observed from the late exponential phase (day 18). (C) Chlorotic subpopulation observed in aging batch cultures of *Prochlorococcus*, belonging to different ecotypes: high-light-adapted MED4 (HLI), MIT9312 (HLII), low-light-adapted NATL2A (LLI), and MIT9313 (LLIV). *Synechococcus* WH8102 is also shown. In all strains, the chlorotic cells begin to emerge at late growth stage, becoming dominant in declining cultures, while in the exponential phase only one population can be observed. Additional growth curves for this strain and for others, including replicates and standard deviations, as shown in Fig. 3 and 4 (see also Fig. S1 to S3, Fig. S5, and Fig. S6 in the supplemental material).

As *Prochlorococcus* batch cultures reach stationary stage and start declining in abundance, the green color of the cultures disappears, and subpopulations of cells emerge with lower chlorophyll autofluorescence that can be identified by flow cytometry (Fig. 1A and B). This phenomenon is observed in strains from all major cultured ecotypes, as well as in a marine *Synechococcus*, strain WH8102 (Fig. 1C). In the high-light adapted strain *Prochlorococcus* MIT9312, lower chlorophyll populations emerged in batch cultures that reached stationary stage due to N or P starvation imposed by the medium composition. However, the timing of the subpopulation emergence and the forward light scatter and chlorophyll autofluorescence (analyzed by flow cytometry) were different under the two nutrient stresses (see Fig. S1A and B in the supplemental material) (33). Cells with lower chlorophyll autofluorescence also appeared in populations of another strain, the low-light-adapted MIT9313, when these cultures were inhibited in a coculture with high cell densities of the heterotrophic bacterium *Alteromonas* sp. strain HOT1A3 (see Fig. S1C and D) (34). Thus, the emergence of populations of cells with lower chlorophyll autofluorescence under a variety of stress conditions is a pervasive phenomenon across marine pico-cyanobacteria. We focused our experiments aiming to better characterize this phenomenon on *Prochlorococcus* sp. strain MIT9313, since the response to stress in this strain has been extensively studied (22, 26, 27, 34–36). In addition, in this strain, three clearly separate subpopulations can be observed when cultured in Pro99 media, facilitating the sorting and subsequent NanoSIMS analyses (Fig. 1B, referred to here as high-, mid-, and low-fl populations).

10.1128/mBio.01846-20.2 FIG S1 Subpopulations of low-chl cells emerge in *Prochlorococcus* cultures under N and P starvation, and when inhibited by heterotrophic bacteria. (A and B) Batch cultures of strain MIT9312 grown under conditions where stationary phase is induced by N and P starvation (A and B, respectively). The data shown are earlier studies (33, 43). (C) Batch cultures of *Prochlorococcus* MIT9313 grown axenically and in coculture with a heterotrophic bacterium, *Alteromonas* HOT1A3. (D) Flow cytometry (FCM) of *Prochlorococcus* populations at two time points (0 and 44 h). The data are from Aharonovich and Sher (34). Download FIG S1, TIF file, 0.7 MB. Copyright © 2020 Roth-Rosenberg et al. 2020 Roth-Rosenberg et al. This content is distributed under the terms of the Creative Commons Attribution 4.0 International license.

### Assessing the metabolic activity of sorted chlorotic subpopulations.

**FIG 2:**
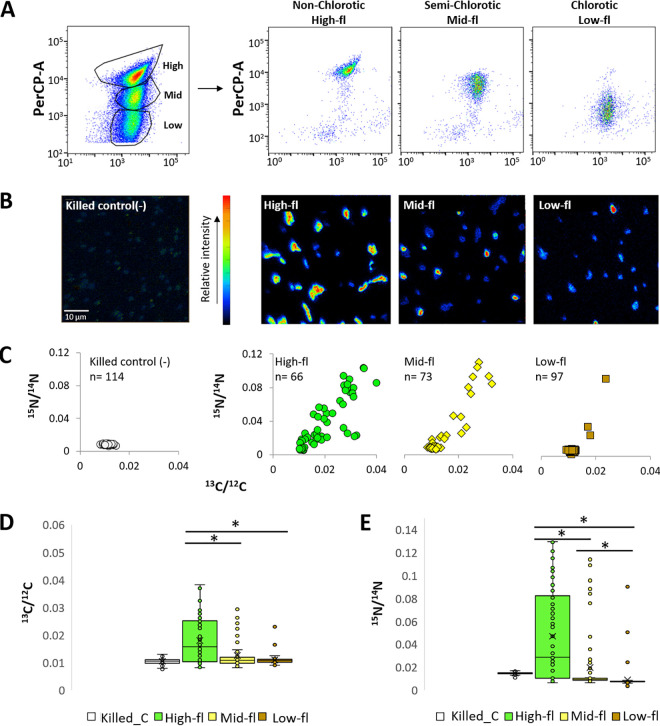
Metabolic activity of subpopulations sorted by NanoSIMS. (A) Flow cytometry scatterplots before and after sorting of three distinct subpopulations (high-, mid-, and low-fl) of an aging *Prochlorococcus* MIT9313 culture, detected by flow cytometry. The cultures were grown for 30 days in Pro99 and labeled with H^13^CO_3_^–^ and ^15^NH_4_^+^ for 18 h. (B) NanoSIMS images of ^15^N/^12^C analysis of killed cells (negative control) and high-, mid-, and low-fl cells after sorting. (C) Scatterplot of ^13^C/^12^C and ^15^N/^14^N ratios obtained from NanoSIMS analysis of each subpopulation. (D and E) Boxplots of the ^13^C/^12^C and ^15^N/^14^N enrichment in each subpopulation. Lines represent the median, X represents the mean, box borders are 1st quartiles, and whiskers represent the full range. Asterisks show significant differences in comparisons between each of the two populations using the Mann-Whitney U test, *P* < 0.001.

We next sought to determine whether the high-, mid-, and low-fl populations differ in their vitality, measured here as their photosynthesis and nutrient uptake rates (incorporation of H^13^CO_3_^–^ and ^15^NH_4_^+^, respectively). The uptake ratio of labeled versus unlabeled nutrients were then used to calculate the metabolic activity of the sorted cells (Table 1). As shown in Fig. 2 and Table 1, the mean uptake of both H^13^CO_3_^–^ and ^15^NH_4_^+^ was highest in the high-fl population, followed by the mid- and low-fl populations, with the latter population indistinguishable from the control, i.e., glutaraldehyde-killed cells (66 to 114 cells were measured [Table 1]; see also Tables S1B and S2). We repeated the entire workflow in an independent experiment, and the results are very similar (see Fig. S2A and B; Table 1, *n* = 88 to 208 cells). These results are reminiscent of observations in several eukaryotic phytoplankton (18). The mean uptake rates for glutaraldehyde killed cells (control) were 0.06 ± 0.15 fg cell^–1^ day^–1^ for C and 0.18 ± 0.02 fg cell^-1^ day^–1^ for N and most likely depict the absorption of the label by nonspecific binding or diffusion.

**TABLE 1:** Calculated mean C and N uptake rates from experiments performed with *Prochlorococcus* MIT9313

Strain, growth stage	Illumination parameters	Subpopulation	No. of cells	Mean *V* (fg cell^−1^ day^−1^)^*d*^ ± SD	
				*V*^ C^	*V*^ N^
MIT9313, exponential growth^*a*^	Constant light, 27 μmol photons m^−2^ s^−1^	High	158	12.92 ± 11.93	2.74 ± 2.43
MIT9313, old cultures^*b*^	Constant light, 27 μmol photons m^−2^ s^−1^	High	118	2.77 ± 3.57	0.62 ± 0.54
			66	2.67 ± 2.88	0.68 ± 0.63
		Mid	208	0.75 ± 1.78	0.32 ± 0.35
			73	0.79 ± 1.73	0.25 ± 0.42
		Low	88	0.09 ± 0.67	0.14 ± 0.12
			97	0.14 ± 0.52	0.08 ± 0.15
MIT9313, old culture^*c*^	Photoperiod: 12:12 L/D, 27 μmol photons m^−2^ s^−1^	High	86	1.01 ± 2.67	0.32 ± 0.33
		Mid	171	0.48 ± 2.47	0.20 ± 0.20
		Low	73	0.13 ± 0.31	0.14 ± 0.11

a The results for MIT9313 exponential growth refer to the experiment presented in Fig. S3 in the supplemental material.

b The results for old MIT9313 cultures under constant light refer to the two experiments presented in Fig. 2 (see also Fig. S2A and B), with the uptake rates and number of cells noted for each experiment separately.

c The results for old MIT9313 cultures under light/dark (L/D) refer to the experiment presented in Fig. S2C to F in the supplemental material. The experiment was sampled after a longer period due to slower growth under a light-dark cycle compared to constant light (Fig. S2C).

d *V*, uptake rate. Means and standard deviations were calculated from the uptake rates determined for single cells in each experiment.

10.1128/mBio.01846-20.3 FIG S2 Independent experiments showing the metabolic activity of sorted subpopulations under constant light and a day/night cycle. (A and B) An independent experiment confirming differences in the metabolic activity of sorted subpopulations of MIT9313 by NanoSIMS. *Prochlorococcus* MIT9313 cultures were grown for 44 days in Pro99 and labeled with H^13^CO_3_^–^ and ^15^NH_4_^+^ for 18 h. (A) Scatterplot of ^13^C/^12^C and ^15^N/^14^N ratios obtained from NanoSIMS analysis of each subpopulation (indicating the number of events detected from multiple fields). (B) Boxplot represent the variances of ^13^C/^12^C and ^15^N/^14^N in each subpopulation. The three populations were statistically different (Kruskal-Wallis test, *P* < 0.001; asterisks show significant differences in comparisons between each of the two populations using the Mann-Whitney U test, *P* < 0.001). (C to F) NanoSIMS analysis for metabolic activity of *Prochlorococcus* chlorotic subpopulations under light/dark growth conditions. For *Prochlorococcus*, cell physiology is strongly entrained by the diel cycle, with cells typically dividing early during the night (38). Since the cultures grown for these experiments presented in Fig. 2 and Fig. S2A and B were grown under conditions of constant light, it was possible that heterogeneity in C and N uptake rates was due to the presence of cells at different cell cycle stages. To test this hypothesis, we repeated this experiment using an MIT9313 culture grown under 12:12 light/dark conditions. (C) MIT9313 growth curve under 12 h light and 12 h dark. (The arrows mark the days shown in panel D.) The dashed line shows the growth under constant light, for comparison. (D) A time series of flow cytometry scattergrams from the tested MIT9313 culture. The *x* axis is forward scatter (FSC; a proxy for cell size); the *y* axis is the chlorophyll autofluorescence of the cells. The appearance of chlorotic subpopulation observed from the late exponential phase (day 31). (E) Scatterplot of ^13^C/^12^C and ^15^N/^14^N ratios obtained from NanoSIMS analysis after 18 h of incubation 3 h L/12 h D/3 h L on day 36. (F) Boxplot of ^13^C/^12^C and ^15^N/^14^N enrichment in each subpopulation. Glutaraldehyde-killed cells were used as a negative control. Lines represent the median, X represents the mean, box borders are 1st quartiles, and whiskers represent the full range. The three populations were statistically different for N uptake (Kruskal-Wallis test, *P* < 0.001) but not for C uptake (*P* = 0.06). Significant differences between each two populations (Mann-Whitney U test) are shown (*, *P* < 0.001). The C uptake rate, as well as the cell-cell heterogeneity, was lower under these conditions, potentially because the cells were in darkness for 12 of the 18 h of labeling, including the “evening” and “morning” periods when the cell’s photosynthetic machinery is not running at its maximal capacity (38). In contrast, the N uptake rate remained high under the light-dark cycle. This suggests that NH_4_ uptake in *Prochlorococcus* is decoupled from photosynthesis and occurs during both light and dark periods, unlike amino acid uptake which occurs in *Prochlorococcus* primarily during the day (57). Download FIG S2, TIF file, 0.9 MB. Copyright © 2020 Roth-Rosenberg et al. 2020 Roth-Rosenberg et al. This content is distributed under the terms of the Creative Commons Attribution 4.0 International license.

10.1128/mBio.01846-20.9 TABLE S1 Calculations of purity and activity of sorted cells. (A) Cell counts and purity of the sorted cells. (B) Number of active and inactive cells in each of the sorted subpopulations. Download Table S1, DOCX file, 0.01 MB. Copyright © 2020 Roth-Rosenberg et al. 2020 Roth-Rosenberg et al. This content is distributed under the terms of the Creative Commons Attribution 4.0 International license.

10.1128/mBio.01846-20.10 TABLE S2 Coefficients of variation. Download Table S2, DOCX file, 0.01 MB. Copyright © 2020 Roth-Rosenberg et al. 2020 Roth-Rosenberg et al. This content is distributed under the terms of the Creative Commons Attribution 4.0 International license.

10.1128/mBio.01846-20.4 FIG S3 NanoSIMS analysis of *Prochlorococcus* MIT9313 from exponentially growing, nutrient-replete cultures compared to late, postdecline stage in batch culture. We measured the N and C uptake rates in MIT9313 cultures for which the stationary stage is induced by N starvation (low-N Pro99, N:P ratio = 2) (43). (A) Growth curve monitored via the chlorophyll autofluorescence. Arrows indicate the heavy-nutrient labeling time points (days 7 and 18). (B) FCM scatterplots of populations of 24 h postlabeling. (C) Scatterplot of ^13^C/^12^C and ^15^N/^14^N ratios obtained from NanoSIMS analysis, representing single cell uptake. (D) Boxplot of ^13^C/^12^C and ^15^N/^14^N enrichment in each population compared to killed cells in the control. Download FIG S3, TIF file, 0.4 MB. Copyright © 2020 Roth-Rosenberg et al. 2020 Roth-Rosenberg et al. This content is distributed under the terms of the Creative Commons Attribution 4.0 International license.

Within each of the populations, cell-cell heterogeneity was observed in both ^13^C and ^15^N uptake (Fig. 2; see also Fig. S2A and B). Within all of the populations (including the high-fl), some cells were inactive, and this could not be explained by the limited purity of the FACS-sorting procedure (see Table S1 and Text S1 in the supplemental material). The coefficients of variation in C and N uptake rates were within the range shown for other organisms, or higher (see Table S2) (32, 37). Similar levels of heterogeneity (primarily in N uptake) were also seen in cells grown under a 12:12 light/dark cycle, where the *Prochlorococcus* cell cycle follows a diel rhythm, suggesting that this heterogeneity is not due to different stages of the cell cycle or the diel cycle (38) (see Table S2 and Fig. S2C to F in the supplemental material). Cell-cell heterogeneity was also observed in cells from an exponentially growing, nutrient-replete culture (see Fig. S3 and Table S2), suggesting that this heterogeneity is not exclusively limited to ageing or stressed cells. This is in accordance with studies assessing the vitality of *Prochlorococcus* cells using various dyes, which consistently show that a significant fraction of the cells in laboratory cultures are inactive or potentially dead (39, 40).

10.1128/mBio.01846-20.1 TEXT S1 Can mis-sorted cells explain the presence of inactive cells in the high-fl population and of active cells in the low- and mid-fl populations? Download Text S1, DOCX file, 0.02 MB. Copyright © 2020 Roth-Rosenberg et al. 2020 Roth-Rosenberg et al. This content is distributed under the terms of the Creative Commons Attribution 4.0 International license.

In addition to differing in their chlorophyll autofluorescence and metabolic activity, the high-, mid-, and low-fl cell populations also differ by their forward and side light scatter properties, which are related to cell size and (in larger cells) morphological complexity (see Fig. S4A and B). In agreement with these observations, cells sorted from the high-fl population and observed by scanning electron microscopy (SEM) were 20 to 30% larger than those from the mid- and low-fl populations (see Fig. S4C to E).

10.1128/mBio.01846-20.5 FIG S4 Sorted cells belonging to different subpopulations of *Prochlorococcus* MIT9313 vary in size. A late-exponential-phase MIT9313 culture was fixed using glutaraldehyde, analyzed using flow cytometry, and the three different subpopulations sorted and observed by SEM. (A and B) Histograms representing changes in cell size (Forward scatter, FSC, panel A) and complexity (side scatter [SSC], panel B) as measured by flow cytometry. (C and D) Boxplots of variations in cell length (C) and cell width (D) as measured from SEM images of sorted populations. High-fl (*n* = 27), mid-fl (*n* = 23), low-fl (*n* = 24) data are shown. The three subpopulations were statistically different (Kruskal-Wallis test, *P* < 0.001). Significant differences between each of the two populations (Mann-Whitney U test) are shown (**, *P* < 0.001; *, *P* < 0.05). Length values from mid-fl and low-fl samples were not significantly different. (E) SEM image of sorted subpopulations. Scale bar, 1 μm. The small square objects in the middle panel are salt crystals. Download FIG S4, TIF file, 0.6 MB. Copyright © 2020 Roth-Rosenberg et al. 2020 Roth-Rosenberg et al. This content is distributed under the terms of the Creative Commons Attribution 4.0 International license.

**FIG 3:**
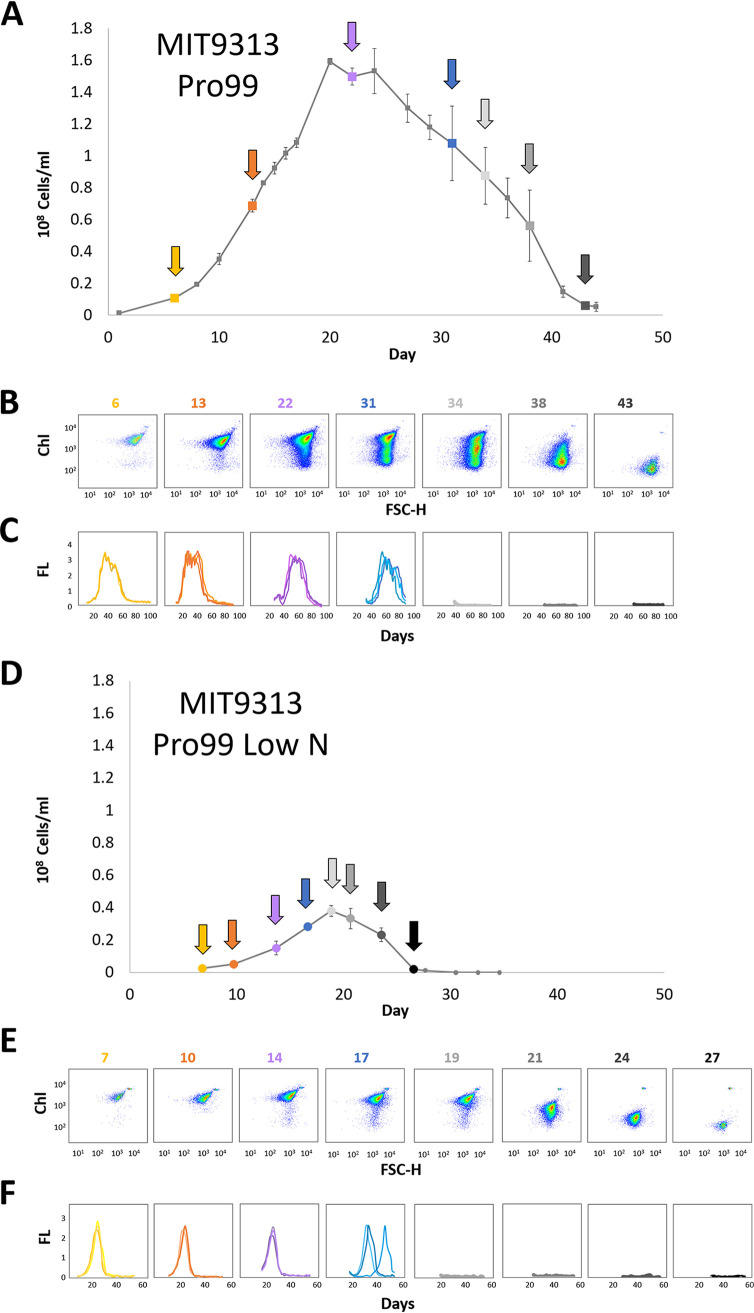
Time-dependent changes in viability of cells transferred into fresh media at different life cycle stages of a batch culture for MIT9313 in Pro99 (A to C) and when stationary stage is induced by N starvation (D to F). (A and D) Growth curves of an MIT9313 culture in Pro99 media commonly used for *Prochlorococcus* culturing (A) and under conditions where stationary stage is induced by nitrogen starvation (D; 2:1 N/P ratio in the growth media) (43). Colored squares/circles indicate the time points at which triplicate 1-ml samples were transferred into 20 ml of fresh media. (B and E) Flow cytometry scatterplots of the culture shown in panels A and D. Note that, under conditions of N starvation, the cultures shift rapidly from being comprised primarily of high-fl cells (day 19 in panel E, early stationary phase) to mainly mid-fl cells, with essentially no high-fl cells (day 21 in panel E). (C and F) Growth curves of cells being transferred at different times to new, nutrient-replete media (assessed via bulk culture fluorescence). In these plots, each line shows a replicate culture. Cells could not regrow when transferred after more than 31 days in Pro99 and 17 days of nitrogen starvation. This suggests that, in this strain, high-fl cells are not necessarily viable.

10.1128/mBio.01846-20.6 FIG S5 Time-dependent changes in viability of *Prochlorococcus* and *Synechococcus* cells from different strains transferred into fresh media at different life cycle stages and different media. In each panel, a growth curve is shown above flow cytometry scatterplots of specific time points (marked by arrows on the growth curves) and growth curves of cells being transferred at different times to new, nutrient-replete media (assessed via bulk culture fluorescence). Error bars on the top curves (cell numbers) represent means and standard deviations of biological triplicates. (A and B) Time-dependent changes in viability of *Prochlorococcus* MIT9312 cells transferred into fresh media at different life cycle stages in Pro99 (A) and when stationary stage is induced by N starvation (B). The experiment in panel A was performed in Pro99 media commonly used for *Prochlorococcus* culturing (A) and in panel B under conditions where stationary stage is induced by nitrogen starvation (2:1 N/P ratio in the growth media) (43). Note that, similar to strain MIT9313 (Fig. 3), under nitrogen starvation, the cultures shift rapidly from being comprised primarily of high-fl cells (day 13 in panel B, early stationary phase) to mainly mid-fl cells, with essentially no high-fl cells (day 15 in panel B). Cells could not regrow when transferred after more than 29 days in Pro99 and 13 days of nitrogen starvation. This suggests that, in this strain, low-fl cells are nonviable. (C) A batch culture of *Prochlorococcus* MIT9313 where stationary stage is induced by P starvation. The media used is Pro99 where P concentrations have been reduced 8-fold, leading to an N/P ratio of 144:1. Cells could not regrow when transferred after more than 17 days. High-fl cells were still seen on days 19 and 21, when the cultures could not be transferred, suggesting that the high-fl cells are not necessarily viable. (D) A marine *Synechococcus*, strain WH 8012, survives much longer under N starvation than *Prochlorococcus*. This experiment was performed in Pro99 media in which N concentrations have been reduced 8-fold, leading to an N/P ratio of 2:1, similar to panel B, above (43). Under these conditions, chlorosis occurs faster than in Pro99, and thus in this experiment high-fl and low-fl were not observed together (compare with Fig. 1C, where the same strain was cultured in Pro99). Two of three cultures survived transfer on day 29 and one of three cultures survived on day 31. Thus, in contrast to all tested *Prochlorococcus* strains, *Synechococcus* WH 8102 could be transferred long after entry into stationary phase, when only low-fl cells were observed. This suggests a fundamentally different relationship between cell chlorophyll fluorescence and viability (the ability to survive culture transfer in this strain). Download FIG S5, TIF file, 1.1 MB. Copyright © 2020 Roth-Rosenberg et al. 2020 Roth-Rosenberg et al. This content is distributed under the terms of the Creative Commons Attribution 4.0 International license.

10.1128/mBio.01846-20.7 FIG S6 Coculture with a heterotrophic bacterium, *Alteromonas* HOT1A3, enables multiple *Prochlorococcus* strains to survive long-term P starvation (A) and when cultured in natural seawater (B). Each panel shows the results of two consecutive experiments. The first experiment is shown on the left side, where either axenic cultures (left column, in green or orange) or cocultures grown with *Alteromonas* HOT1A3 (right column, grey lines) are inoculated into the tested media. After 40 days, 1ml from each culture was transferred into fresh Pro99 media (right side of each panel), and the new cultures were monitored for an additional 50 days. In these plots, each line shows a replicate culture. (A) Culture survival under P starvation. PO_4_ concentration in the media was set to 6.25μM, leading to an N:P ratio of 128 rather than 16). Note the differences in the units on the y-axes. The length of starvation is longer in this experiment compared with the one presented in Fig. S5D (where *Synechococcus* WH 8102 cultures grew when transferred into new media for up to 31 days in N-starved cultures). (B) Culture survival in natural seawater (filtered but with no nutrients or trace metals added). Growth under these conditions is likely due to nutrient carryover from the original cultures, which is less than 40 μM NH_4_ and 2.5 μM PO_4_ (cultures were not centrifuged and resuspended in seawater to avoid cell stress). Note that, under these conditions, growth of *Prochlorococcus* in coculture was reduced, potentially due to competition for inorganic nutrients by *Alteromonas*. Download FIG S6, TIF file, 0.6 MB. Copyright © 2020 Roth-Rosenberg et al. 2020 Roth-Rosenberg et al. This content is distributed under the terms of the Creative Commons Attribution 4.0 International license.

### Evaluating the viability of subpopulations.

We next sought to determine whether the low-fl cells are viable resting stages. We tested this indirectly by determining the ability of *Prochlorococcus* MIT9313 cells, cultured in Pro99 media, to grow upon transfer to new growth media at different times during exponential growth and upon culture decline. As shown in Fig. 3A, only cells from cultures where the high-fl cells were dominant could grow when transferred to new growth media. No growth was observed upon transfer of cells from stationary or declining cultures where no high-fl cells were observed. Intriguingly, the presence of high-fl cells was not enough to ensure culture growth (e.g., day 34 in Fig. 3A). This is consistent with a previous study showing that cells belonging to a different *Prochlorococcus* strain, MED4, that were incubated for 3 days in the dark were unable to resume growth after return to light despite showing no clear difference in the chlorophyll autofluorescence (30). The probability of growth after transfer did not depend on the number of transferred cells (41), with as many as 2.5 × 10^7^ cells/ml failing to grow after transfer during culture decline (cells at ∼1/10 of this density grew after being transferred during exponential stage). Thus, nonchlorotic cells (defined as being within the range of chlorophyll autofluorescence exhibited by exponentially growing cells) are not necessarily viable.

One problem with performing experiments in Pro99, which is commonly used to culture *Prochlorococcus*, is that the conditions causing cells to reach stationary stage are not always clear (e.g., (42). We therefore repeated these experiments under conditions where entry into stationary phase is induced by N or P starvation (Fig. 3B; see also Fig. S5) (43). When entry into stationary stage was induced by N or P starvation, chlorotic cells appeared much faster, and the cultures became nonviable much earlier, i.e., essentially immediately after the cessation of exponential growth. Similar results were obtained with a different strain of *Prochlorococcus*, MIT9312 (see Fig. S5A and B). However, a marine *Synechococcus* strain (WH8102) behaved differently, surviving N starvation much longer and being able to regrow in nutrient-replete media long after the culture started declining and when essentially all cells were chlorotic (see Fig. S5D). This is reminiscent of the ability of (presumably axenic) cultures of two freshwater cyanobacteria, *Synechococcus elegantus* PCC 7942 and *Synechocystis* PCC 6803, to revive after extended N starvation (14, 15).

**FIG 4:**
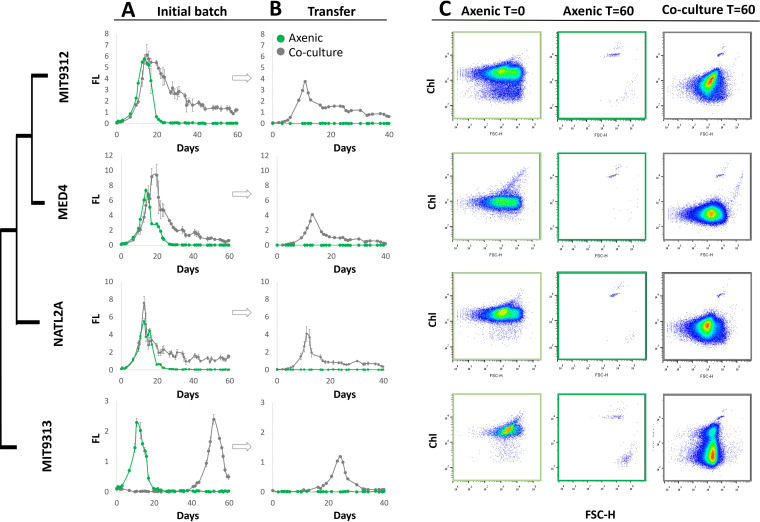
Coculture with a heterotrophic bacterium, *Alteromonas* HOT1A3, enables multiple *Prochlorococcus* strains to survive long-term N starvation. (A) 10^6^ axenic *Prochlorococcus* cells ml^−1^ from different strains were incubated alone (green line) or with the addition of 10^7^ *Alteromonas* HOT1A3 cells ml^−1^ in low-N media (gray line). Bulk culture fluorescence was recorded as a proxy for cell growth, and 1 ml from each culture was transferred into 25 ml of fresh Pro99 media after 60 days. (B) The transferred cultures were recorded for additional 40 days. Error bars are standard deviations from triplicate cultures. The late growth of MIT9313 in coculture is the “delayed growth” phenotype described previously (34, 45). (C) Flow cytometry scattergrams of the cultures shown in panel A. Coculture with *Alteromonas* increases the number of *Prochlorococcus* cells seen by flow cytometry, suggesting a reduction in the magnitude of mortality (cell lysis). A decrease in the per-cell chlorophyll autofluorescence is still seen, suggesting coculture does not completely inhibit the process of chlorophyll degradation.

The inability of axenic *Prochlorococcus* strains to survive long-term nutrient starvation was surprising, and we therefore hypothesized that their survival would be enhanced by interactions with co-occurring heterotrophic bacteria. Indeed, when cocultured with a heterotrophic bacterium, *Alteromonas* HOT1A3 (34, 44), *Prochlorococcus* strains representing all major cultured ecotypes were able to regrow after 60 days of N and P stress, whereas all axenic strains failed to do so (Fig. 4; see also Fig. S6A and B in the supplemental material). Interestingly, strain MIT9313, which was initially inhibited by this *Alteromonas* strain (Fig. 4A) (34, 45), was also able to survive long-term starvation in coculture, suggesting that fundamentally different interactions occur during exponential growth compared to long-term, presumably nutrient-limited growth. These results are consistent with the ability of heterotrophic bacteria to extend the survival time of different *Prochlorococcus* strains under conditions of constant darkness (albeit for only several days [30]) and with the ability of different heterotrophic bacteria to support the long-term viability of batch cultures of *Synechococcus* WH7803 (46).

It has previously been suggested that detoxification of waste products may be one mechanism whereby heterotrophic bacteria support the long-term survival of pico-cyanobacteria (46). To test this, we transferred *Prochlorococcus* cultures from nutrient-replete media into sterile seawater with no added nutrients (see Fig. S6B). Under these conditions, *Prochlorococcus* reach much lower densities than in laboratory media (∼10-fold lower fluorescence, particularly in coculture, compare Fig. S6B and, e.g., Fig. 4), and thus it is unlikely that they are inhibited by their own waste products. Although coculture with *Alteromonas* HOT1A3 reduced the growth of all *Prochlorococcus* and *Synechococcus* strains under these conditions, potentially due to competition for scarce nutrients, only the cocultures were able to survive and grow when transferred into fresh media after 40 days (right panel in Fig. S6B). We therefore hypothesize that the ability of the heterotrophs to support *Prochlorococcu*s survival in extended coculture is due to nutrient remineralization rather than to the detoxification of potential waste products, although a potential role for detoxification cannot be ruled out.

### Stress survival in pico-cyanobacteria: why is *Prochlorococcus* different?

In the present study, we demonstrate that phenotypic heterogeneity between clonal *Prochlorococcus* cells occurs at multiple “scales.” In exponentially growing axenic laboratory cultures of two strains, MIT9313 and MED4, C and N uptake rates differ significantly between individual cells (summarized in Table S2 in the supplemental material). This variation is independent of genetic variability. In addition, as axenic cultures become stressed, a larger phenotypic change occurs as cells lose their chlorophyll auto-fluorescence and become chlorotic. Under these experimental conditions, most cells are inactive (primarily in the low-fl population, as measured in strain MIT9313), although we cannot rule out that even low-fl cells still retain a residual level of activity not detectable by the NanoSIMS. Nevertheless, some cells from the chlorotic populations retain at least part of their photosynthetic capacity and indeed can fix carbon and take up NH_4_. Yet, in our experiments, they do not regrow when conditions become more favorable. In *Synechococcus elegantus* PCC 7942, chlorotic cultures retain approximately 0.01% of their photosynthetic activity, as well as a residual level of protein translation, although it remains unclear whether this is a process shared by all cells in the culture or whether this activity is only due to a small subset of more active cells (14). The clear difference between the ability of axenic *Synechococcus elegantus* PCC 7942 and *Synechocystis* PCC 6803 to survive long-term N starvation, as well as the inability of axenic *Prochlorococcus* cultures to do so, suggests an inherent difference in the physiology and genomic functional capacity between these unicellular cyanobacteria.

Entry into chlorosis in *Synechocystis* is a regulated process that involves the organized degradation of the phycobilisomes in parallel with an increase in the storage products glycogen and polyhydroxybutyrate (PHB) (15). The photosynthesis apparatus of *Prochlorococcus* is different from that of other cyanobacteria, using unique chlorophyll *a*_2_/*b*_2_ binding proteins rather than phycobilisomes (47). Indeed, *Prochlorococcus* lack orthologs of the *nblA* gene required for phycobilisome degradation during chlorosis (15). *Synechococcus* WH8102, which was able to survive N starvation much longer in axenic culture than the tested *Prochlorococcus* strains (Fig. S5D), has a divergent *nblA*-like gene (48). In addition, while *Prochlorococcus* likely use glycogen as a C storage pool (49), they lack the *phaA*, *phaB*, *phaC*, and *phaE* genes required for PHB biosynthesis which are induced in *Synechocystis* PCC 6803 under chlorosis (although these genes are not required for revival from chlorosis [15]). Taken together, these differences suggest that *Prochlorococcus* lack the genetic toolkit employed by *Synechocystis* PCC 6803 and *Synechococcus elegantus* PCC 7942 to enter into a resting stage. Thus, chlorotic cells in *Prochlorococcus* are not resting stages.

If *Prochlorococcus* are indeed incapable of producing resting stages in response to nutrient or light starvation, what are the evolutionary drivers of this phenotype, and what are the consequences for the dynamics of *Prochlorococcus* populations in the ocean? While the open oligotrophic ocean is often considered a relatively stable environment, nutrient concentrations do fluctuate (8), and phytoplankton (including *Prochlorococcus*) inhabiting these waters show multiple signs of nutrient stress (50). Many of the microbes that live in such environments comprising a large fraction of the surface ocean have small, highly streamlined genomes (51), and this has been suggested to be an adaptation to low nutrient concentrations (24, 51, 52). Therefore, it is possible that the lack of resting stages is a result of this genome streamlining: the genomes of *Synechococcus elegantus* PCC 7942 and *Synechocystis* PCC 6803 are ∼3.2 and ∼4 Mbp with their plasmids, respectively, compared to ∼1.4 to 2.5 Mbp for *Prochlorococcus* strains.

### Surviving nutrient stress “with a little help from my friends.”

The ability of *Prochlorococcus* to thrive under conditions of extreme nutrient limitation is often explained by their small cell size (increasing their biomass-specific diffusion), their generally low nutrient requirements, and their specific metabolic strategies to minimize the per-cell elemental quotas (53–55). However, these mechanisms appear not to work in axenic laboratory cultures, and thus we propose that interactions with co-occurring microorganisms enable *Prochlorococcus* to survive when nutrient-saving mechanisms are not sufficient, as suggested for its close relative *Synechococcus* (46). This may take the form of recycling of inorganic nutrients by the heterotrophic bacteria, as well as possibly by the production of organic compounds that contain elements such as N or P. Indeed, *Prochlorococcus* can compete with heterotrophic bacteria for amino acids (56, 57). Importantly, the utilization of organic compounds (mixotrophy) may provide *Prochlorococcus* also with carbon, sulfur, or energy sources and may potentially help them survive also light starvation (30, 58–62).

Regardless of the specific forms of dissolved organic matter being utilized by the cells, and on the exact mechanism enabling the cells to survive long-term nutrient starvation in coculture, the lack of any observed mechanism for the production of resting stages by *Prochlorococcus* may be considered another manifestation of the “black queen hypothesis.” This hypothesis states that microorganisms “outsource” essential survival mechanisms such as detoxification of reactive oxygen species to the surrounding microbial community (63). These forms of microbial interactions likely affect the distribution and activity of *Prochlorococcus* on a global scale. The increased survival of *Prochlorococcus* under harsh conditions, supported by its associated heterotrophic bacteria, may enable it to remain active at the single cell level even during long periods of unfavorable conditions (64, 65). Thus, the tight interactions between *Prochlorococcus* and its bacterial “supporters” likely affects photosynthesis and carbon cycling at the base of the aquatic food web, with potentially profound implications for overall oceanic productivity and carbon cycling.

## MATERIALS AND METHODS

### *Prochlorococcus* growth and stable isotope incubations.

Axenic *Prochlorococcus* strains were grown in Pro99 media under constant cold while light (27 μmol photons m^−2^ s^−1^) at 22°C. Axenicity of the strains was tested routinely, as well as before all major experiments, using test media (ProMM [41]). In addition, no evidence for contaminating heterotrophic cells was observed using flow cytometry or SEM or when axenic *Prochlorococcus* cultures were used as negative controls for 16S amplicon sequencing. Bulk chlorophyll fluorescence (excitation, 440 nm; emission, 680 nm) was measured almost daily using a fluorescence spectrophotometer (Cary Eclipse; Varian). In parallel, samples for flow cytometry were taken for cell numbers. When three distinct subpopulations appeared in the flow cytometry, the cultures were labeled with 1 mM Sodium bicarbonate-^13^C and 1 mM ammonium-^15^N chloride (Sigma-Aldrich) for 18 to 24 h. The optimal incubation time is based on preliminary isotope labeling experiments with *Prochlorococcus* MED4, showing that uptake is identified already after 3 h and is linear until 24 h under our growth conditions (see Fig. S7). Incubations were stopped by fixing 2 ml of the culture with 2× EM-grade glutaraldehyde (2.5% final concentration; Sigma) and subsequent storing at 4°C until the sorting analysis. Nonlabeled cells that were killed before labeling (by adding 2.5% glutaraldehyde) were used as a negative control.

10.1128/mBio.01846-20.8 FIG S7 Optimization for measuring metabolic activity of *Prochlorococcus* by NanoSIMS (A) Growth curve of *Prochlorococcus* MED4 measured by fluorescence 440/680nm. Labeled nutrients (H^13^CO_3_^–^ and ^15^NH_4_^+^) were added after day 6 and sampled for NanoSIMS at 3, 6, 12, and 24 h. Insert shows the cell population at the time of labeling (T0) by FCM. (B) NanoSIMS images of ^15^N/^12^C analysis of cell for measuring metabolic activity. (C) Scatterplot of ^13^C/^12^C and ^15^N/^14^N ratios obtained from NanoSIMS analysis at each time point (indicating the number of events detected from multiple fields). (D) Boxplots represent the variances of ^13^C/^12^C and ^15^N/^14^N at each time point. Download FIG S7, TIF file, 0.8 MB. Copyright © 2020 Roth-Rosenberg et al. 2020 Roth-Rosenberg et al. This content is distributed under the terms of the Creative Commons Attribution 4.0 International license.

### Cell sorting and filtration.

Sorting of subpopulation was carried out using a BD FACSAria III sorter (BD Biosciences) at the Life Sciences and Engineering Infrastructure Center, Technion, Israel. Each sample was sorted for three subpopulations: nonchlorotic (high-fl), semichlorotic (mid-fl), and chlorotic (low-fl) (Fig. 2A). The sorting gates for each subpopulation were determined from the population observed in forward scatter (FSC; a proxy for cell size) and autofluorescence (PerCP, chlorophyll autofluorescence). After sorting, the sorted subpopulation was gently filtered on 13-mm-diameter polycarbonate filters (GTTP, 0.2-μm pore size; Millipore, MA), washed twice with sterile seawater, and air dried. The filters were cut into two parts. One half was stored at 4°C until NanoSIMS analyses, and the other half is used for SEM.

### NanoSIMS and data analysis.

The samples were coated with a layer of ∼30-nm gold with a Cressington 108 auto sputter coater (Watford, United Kingdom). Random spots were used for NanoSIMS analyses. SIMS imaging was performed using a NanoSIMS 50L instrument (Cameca, Paris, France) at the Leibniz-Institute for Baltic Sea Research Warnemünde (IOW). A ^133^Cs^+^ primary ion beam was used to erode and ionize atoms of the sample. Images of secondary electrons—^12^C^–^, ^13^C^–^, ^12^C^14^N, and ^12^C^15^N^–^—were recorded simultaneously using mass detectors equipped with electron multipliers (Hamamatsu). The mass resolving power was adjusted to be sufficient to suppress interferences at all masses allowing, e.g., the separation of ^13^C^–^ from interfering ions such as ^12^C^1^H^–^. Prior to the analysis, sample areas of 50 × 50 μm were sputtered for 2 min with 600 pA to erode the gold, clean the surface, and reach the steady state of secondary ion formation. The primary ion beam current during the analysis was 1 pA; the scanning parameters were 512 × 512 pixels for areas of 30 × 30 to 48 × 48 μm, with a dwell time of 250 μs per pixel. A total of 60 planes were analyzed.

### Analyses of NanoSIMS measurements.

All NanoSIMS measurements were analyzed with the Matlab based program look@nanosims (66). Briefly, the 60 measured planes were checked for inconsistencies and all usable planes accumulated, regions of interest (ROIs) (i.e., *Prochlorococcus* cells and filter regions without organic material for background measurements) defined based on ^12^C^14^N mass pictures, and both ^13^C/^12^C and ^12^C^15^N/^12^C^14^N ratios calculated from the ion signals for each ROI.

### Uptake rate calculation.

Uptake rate was estimated using the following equation, based on that of Legendre and Gosselin (67), as follows:

$$V=\frac{\left(\%P_{t}^{*}-\%P_{0}^{*}\right)}{\left(\%D_{i}^{*}-\%D_{0}^{*}\right)}\frac{Q}{t}$$

Where $\%P_{t}^{*}$ is the concentration (atom %) of the heavy isotope in the particulate matter at the end of the incubation, $\%D_{i}^{*}$ is the concentration of the dissolved tracer added to the incubation (and assumed not to change over the short incubation time), and $\%P_{0}^{*}$and $\%D_{0}^{*}$ are the natural heavy isotope concentrations in the particulate and dissolved matter, respectively. We estimated *Q*, the cell quota (in fg cell^−1^) of C or N, based on measurements of the biomass of MED4 and MIT9313 (66 and 158 fg cell^−1^, respectively [68]) and assuming that C comprises 50% and N comprises 7.5% of the cell biomass. For heavy isotopes concentration in the particulate and dissolved phases before incubation we used the natural values for isotopic ratios of ^13^C and ^15^N (1.12 and 0.37%, respectively). For the experiment shown in Fig. S7 in the supplemental material, we measured the NH_4_^+^ concentration in the media and added the ^15^N tracer to 50% final concentration. Since all other experiments were performed in declining cultures, we assumed that the NH_4_^+^ was depleted from the media, and thus $\%D_{t}^{*}$ was defined as 90%, based on previous measurements of NH_4_^+^ concentrations in old cultures. We used a value of 50% for the initial percentage of ^13^C, based on dissolved inorganic carbon (DIC) measurements (43). For the terminal concentrations of ^15^N and ^13^C in the particulate phase ($\%P_{t}^{*}$), we used the values of ^13^C/^12^C and ^15^N/^14^N that were obtained from the NanoSIMS analysis of the cells. ^13^C/^12^C and ^15^N/^14^N ratios below the natural values resulted in negative uptake values and were treated as zero uptake.

Means and standard deviations (SD) of C and N uptake rates were calculated from the uptake rate values of individual cells (Table 1). The uptake rate values were not corrected for negative control (killed cells), which are presented for comparison in Table 1. Since ^13^C/^12^C and ^15^N/^14^N values of individual cells were not normally distributed, for significance analysis we used nonparametric tests (Mann-Whitney and Kruskal-Wallis tests) performed using the real statistics resource pack software (release 5.4).

### Scanning electron microscopy.

Immediately after filtration, filters dehydrated in an ethanol series of 30, 50, 70, 80, 90, and 100% (vol/vol) ethanol (dilutions were in deionized water) for 10 min each. Samples were then dried, mounted on stubs with carbon tape, and coated with 5-nm gold. Cells were obtained on a Zeiss Sigma SEM by using a SE2 detector (2 to 2.5 kV, WD = 8 mm).
