# Supplementary figures and images for: Prochlorococcus Cells Rely on Microbial Interactions Rather than on Chlorotic Resting Stages To Survive Long-Term Nutrient Starvation
Source: mBio. 2020 Aug 11;11(4):e01846-20. doi: 10.1128/mBio.01846-20 (PMC7439483; doi:10.1128/mBio.01846-20)

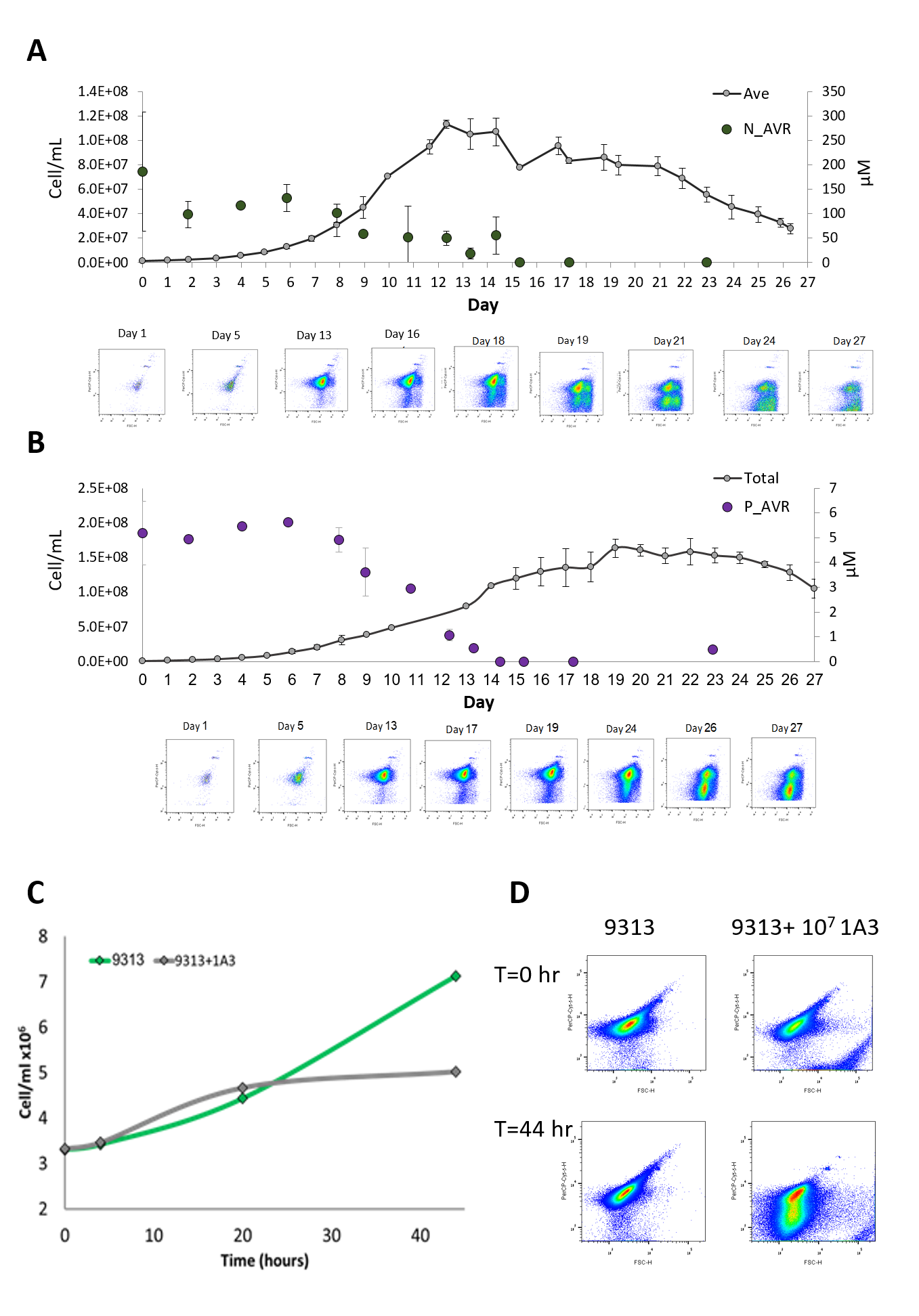

Supplement: FIG S1 [file mBio.01846-20-sf001.tif]

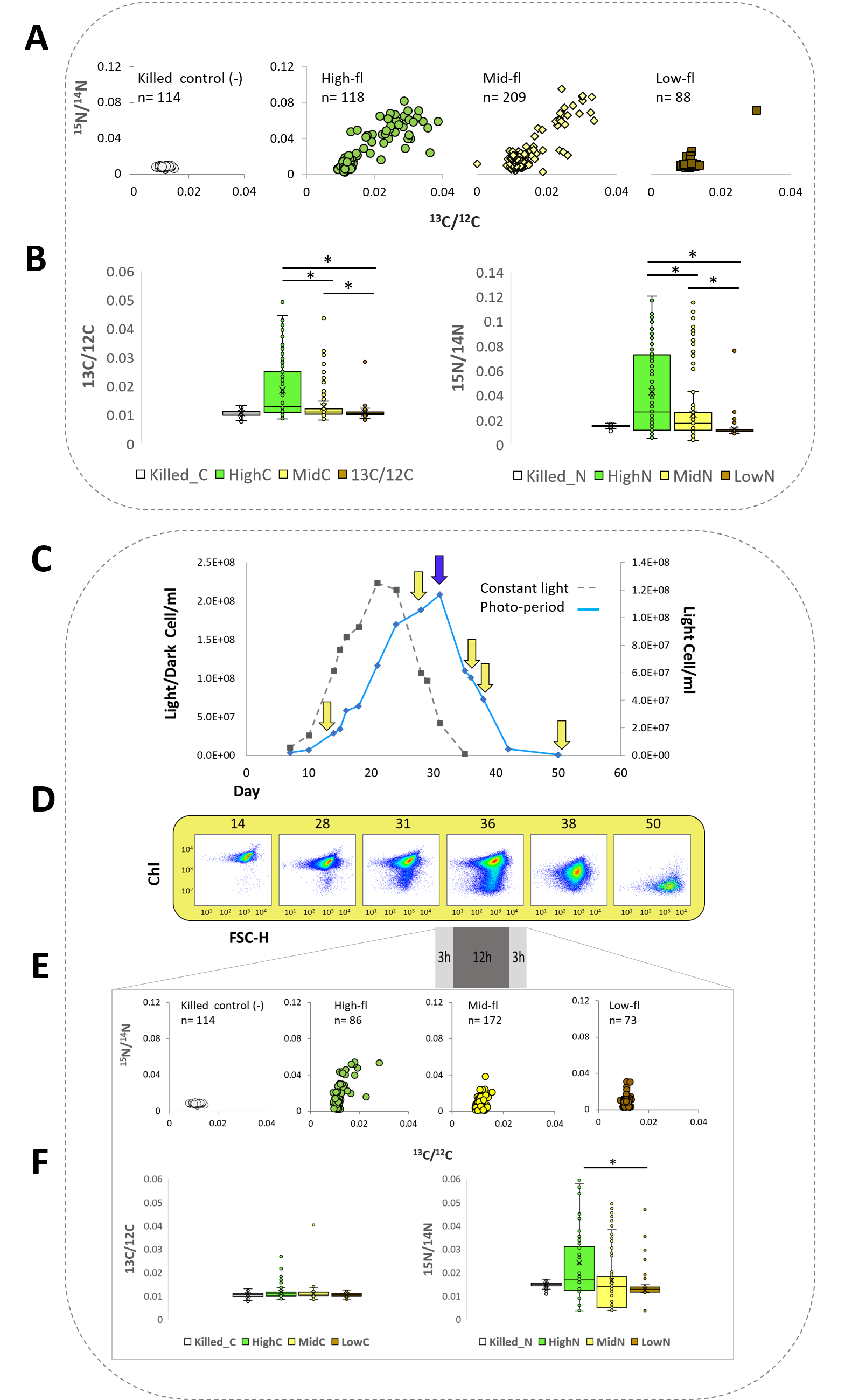

Supplement: FIG S2 [file mBio.01846-20-sf002.tif]

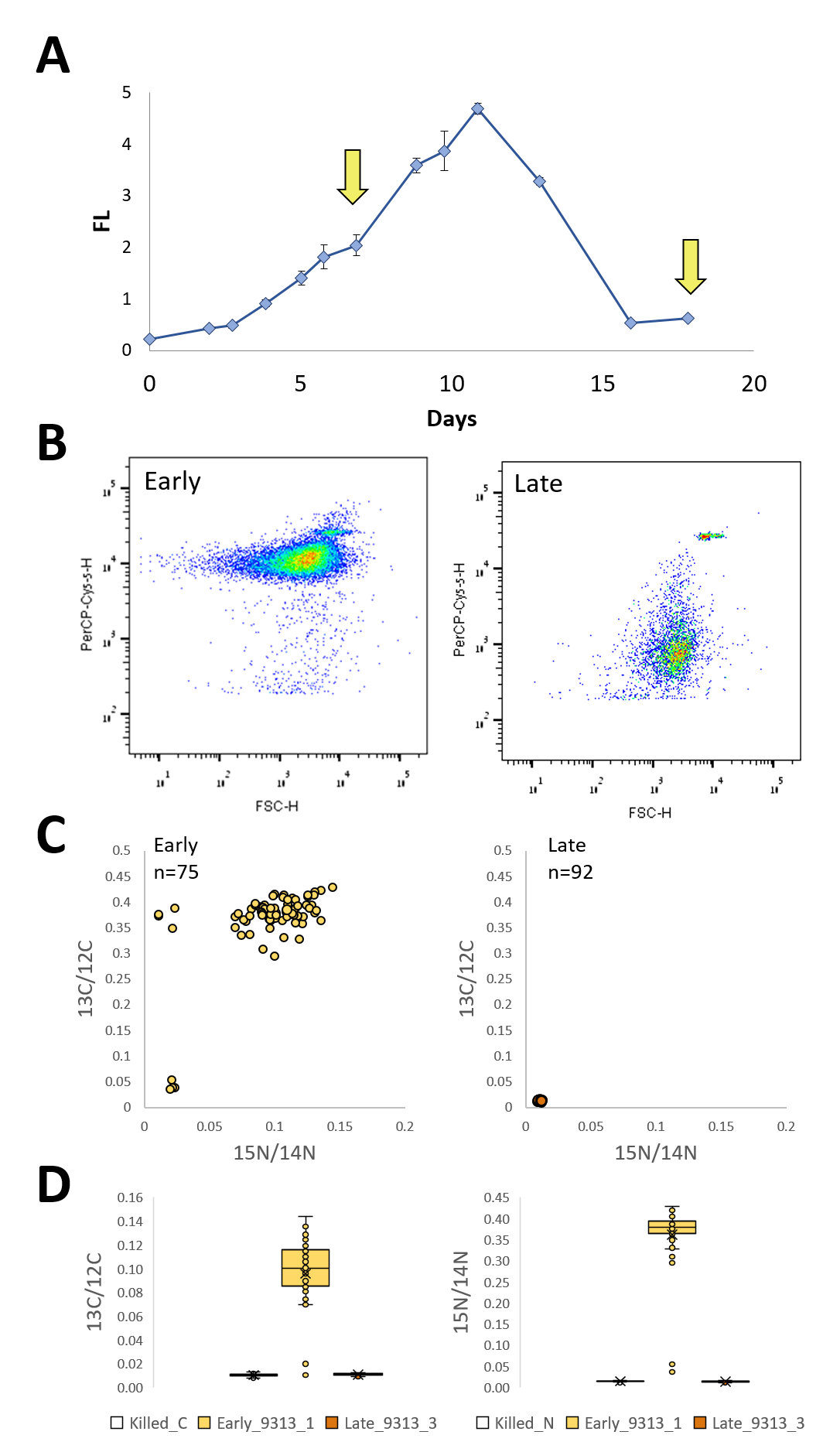

Supplement: FIG S3 [file mBio.01846-20-sf003.tif]

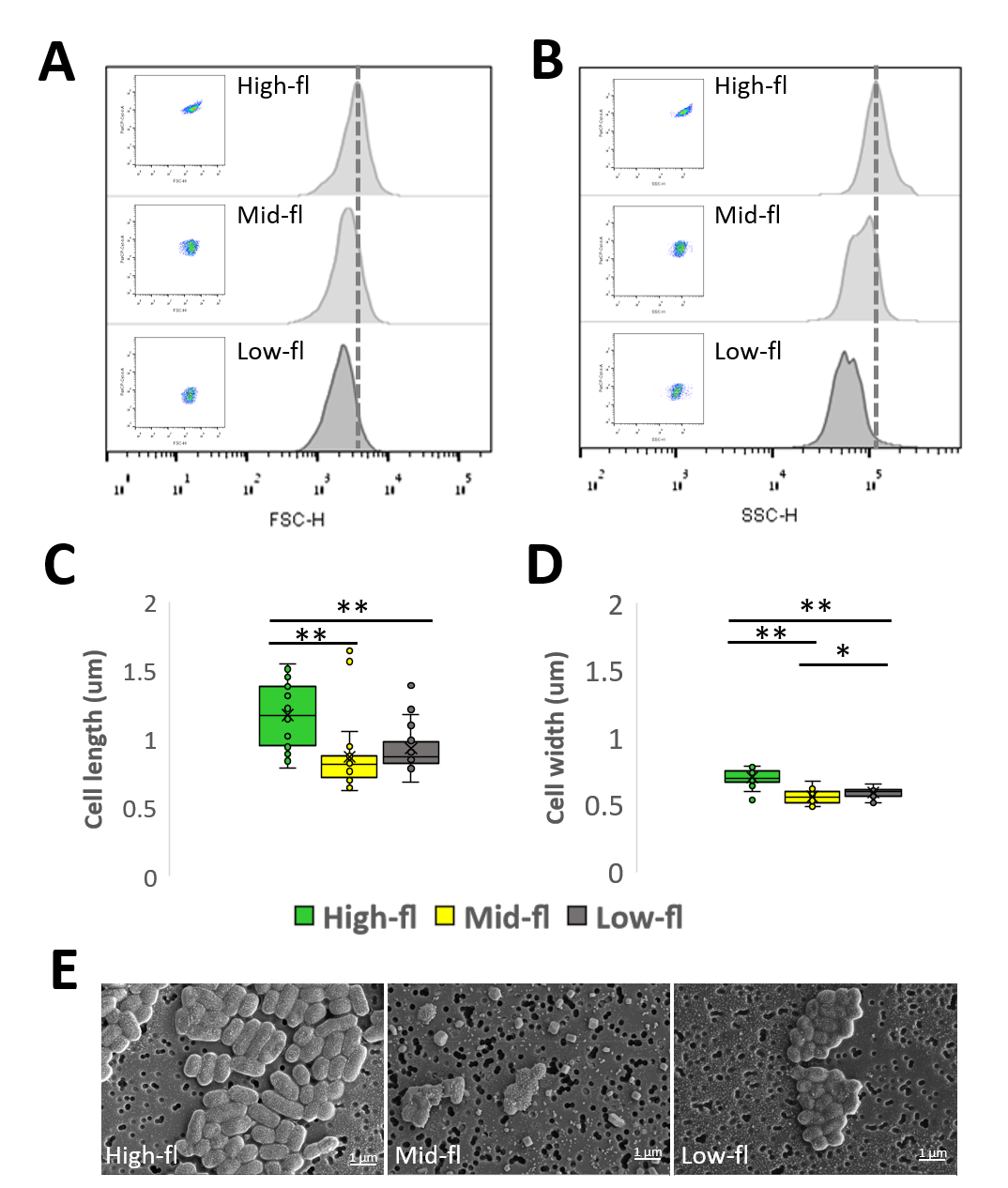

Supplement: FIG S4 [file mBio.01846-20-sf004.tif]

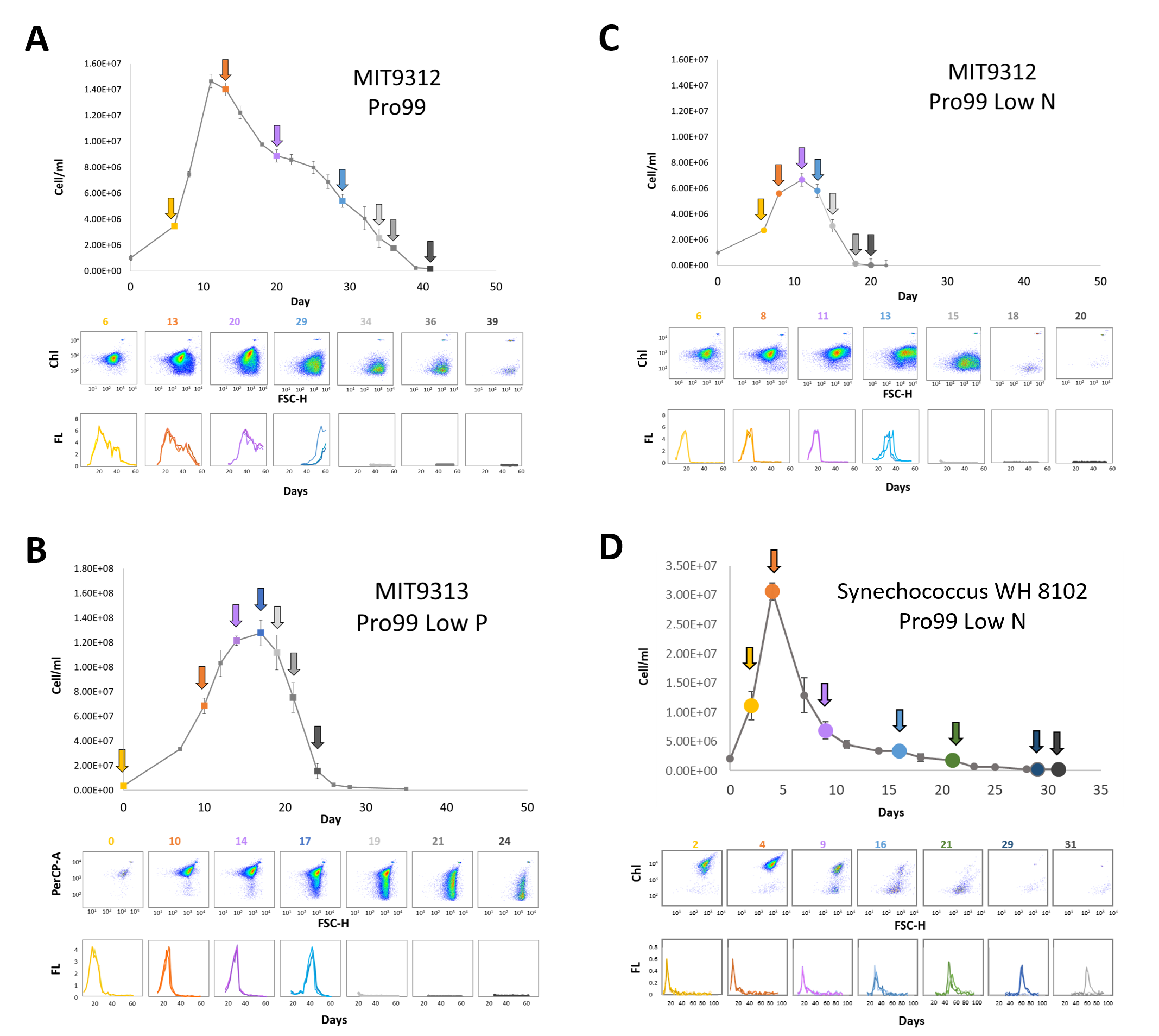

Supplement: FIG S5 [file mBio.01846-20-sf005.tif]

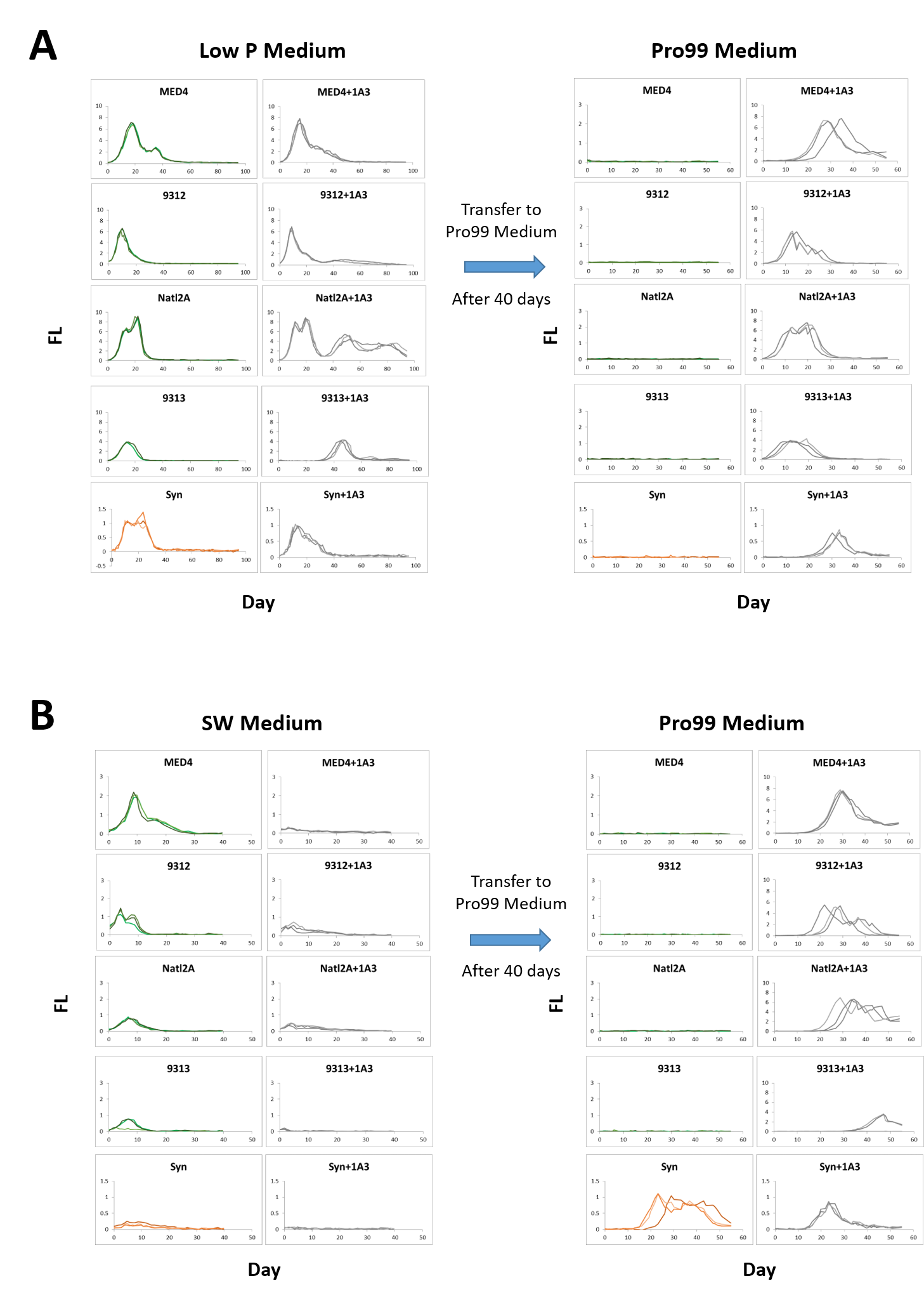

Supplement: FIG S6 [file mBio.01846-20-sf006.tif]

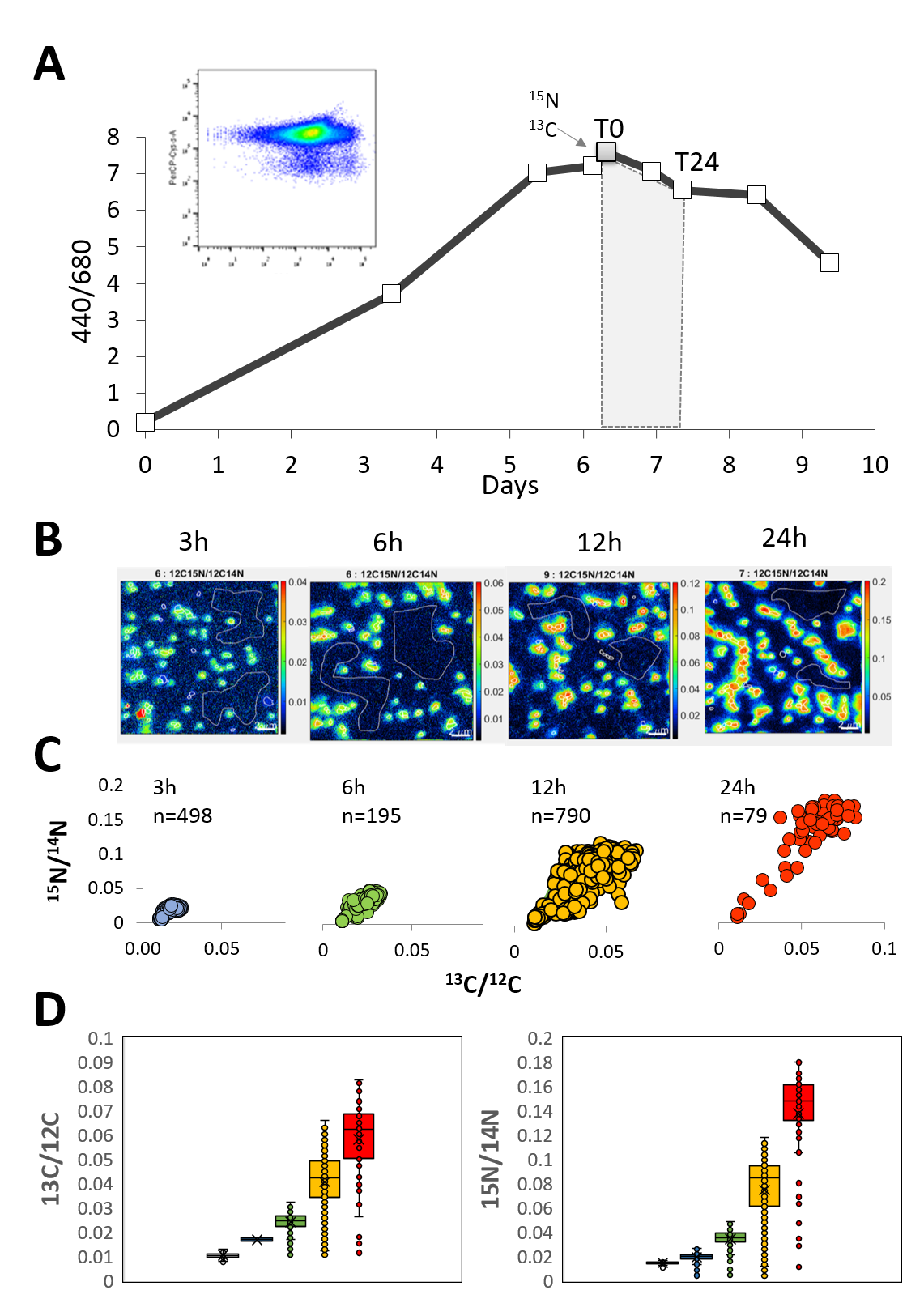

Supplement: FIG S7 [file mBio.01846-20-sf007.tif]
