# Supplementary material for: Prochlorococcus Cells Rely on Microbial Interactions Rather than on Chlorotic Resting Stages To Survive Long-Term Nutrient Starvation
Source: mBio. 2020 Aug 11;11(4):e01846-20. doi: 10.1128/mBio.01846-20 (PMC7439483; doi:10.1128/mBio.01846-20)
Supplement: TABLE S1 [file mBio.01846-20-st001.docx]

**Supplementary Table S1A: Cell counts and purity of the sorted cells**

|  |  | Sorted sub-populations  *MIT9313 old | | | Sorted sub-populations  **MIT9313 old | | |
| --- | --- | --- | --- | --- | --- | --- | --- |
| **% Purity and number of sorted cells** |  | **High** | **Mid** | **Low** | **High** | **Mid** | **Low** |
|  | **High** | 3388 (94%) | 239 (7.9%) | 19  (1%) | 3109 (84.5%) | 301 (7.5%) | 100 (2.9%) |
|  | **Mid** | 174 (4.8%) | 2673 (88.2%) | 172 (9.1%) | 514 (14%) | 3639 (91%) | 685 (19.6%) |
|  | **Low** | 59 (1.6%) | 118 (3.9%) | 1699 (90%) | 56  (1.5%) | 57 (1.4%) | 2715 (77.6%) |
|  | **Total cells** | 3621 | 3030 | 1890 | 3621 | 3030 | 1890 |

*The result refers to the experiment presented in Fig. 2

** The result refers to the experiment presented in Fig S2A,B.

**Supplementary Table S1B: Number of active and inactive cells in each of the sorted sub-populations.**

|  | Sorted sub-populations  *MIT9313 old | | | Sorted sub-populations  **MIT9313 old | | |
| --- | --- | --- | --- | --- | --- | --- |
|  | **High** | **Mid** | **Low** | **High** | **Mid** | **Low** |
| % and number of active cells | 45  (68%) | 16  (22%) | 3  (3.1%) | 75  (64%) | 107  (51%) | 5  (6%) |
| % and number of inactive cells | 21  (31.8%) | 57  (78%) | 94  (97%) | 43  (36%) | 101  (49%) | 83  (94%) |
| Total cells | 66 | 73 | 97 | 118 | 208 | 88 |

*The result refers to the experiment presented in Fig. 2

** The result refers to the experiment presented in Fig S2A,B
