# Supplementary material for: Prochlorococcus Cells Rely on Microbial Interactions Rather than on Chlorotic Resting Stages To Survive Long-Term Nutrient Starvation
Source: mBio. 2020 Aug 11;11(4):e01846-20. doi: 10.1128/mBio.01846-20 (PMC7439483; doi:10.1128/mBio.01846-20)
Supplement: TABLE S2 [file mBio.01846-20-st002.docx]

**Supplementary Table S2:** **Coefficient of variation**

| **Strain and growth stage** | | **Sub-population** | **Illumination** | **Number of cells** | **C_cov_** | | **N_cov_** |
| --- | --- | --- | --- | --- | --- | --- | --- |
| **Batch culture** | |  |  |  | |  |  |
| MIT9313, exponential growth* | | High | Constant light  27  μmol photons m-2 s-1 | 158 | | 0.92 | 0.89 |
| MIT9313,  old culture** | | High |  | 66-118 | | 1.08-1.29 | 0.92-0.87 |
|  |  | Mid |  | 73-208 | | 2.19-2.37 | 1.63-1.09 |
|  |  | Low |  | 88-97 | | 3.77-7.11 | 1.83-0.85 |
| MIT9313,  old culture*** | | High | Photo-period  12:12 L/D, 27  μmol photons m-2 s-1 | 86 | | 2.65 | 1.02 |
|  |  | Mid |  | 171 | | 5.15 | 0.99 |
|  |  | Low |  | 73 | | 0.69 | 2.33 |
| Med4  Exponential growth**** | 3h |  | Constant light 27  μmol photons m-2 s-1 | 489 | 0.40 | | 0.25 |
|  | 6h |  |  | 189 | 0.31 | | 0.25 |
|  | 12h |  |  | 780 | 0.39 | | 0.38 |
|  | 24h |  |  | 77 | 0.32 | | 0.28 |
| **Killed cells** | |  |  | 114 | | 2.63 | 0.09 |

*The results for MIT9313 exponential growth refer to the experiment presented in Fig. S3.

**The results for old MIT9313 cultures under constant light refer to the two experiments presented in Fig. 2 and Fig. S2A, B.

*** The results for Old MIT9313 cultures under L/D refer to the experiment presented in Fig. S2C-F.

**** The results for MED4 cultures refer to the experiment presented in Fig. S7.

For comparison, COVs of ~0.5-0.6 were recorded for uptake of different 15N-labeled N sources by the dinoflagellate *Prorocentrum minimum* (Matantseva et al, Frontiers in Microbiology. 7:1310, 2016). Significantly larger variation was observed in field populations of photosynthetic picoeukaryotes, *Synechococcus* and *Prochlorococcus*, with *Prochlorococcus* revealing a COV of ~0.2-0.4 for ^13^CO_2_ and ~0.1-0.7 for ^15^NH_4_ (Berthelot et al, ISMEJ 13:651-662, 2019)
