# Supplementary material for: Prochlorococcus Cells Rely on Microbial Interactions Rather than on Chlorotic Resting Stages To Survive Long-Term Nutrient Starvation
Source: mBio. 2020 Aug 11;11(4):e01846-20. doi: 10.1128/mBio.01846-20 (PMC7439483; doi:10.1128/mBio.01846-20)
Supplement: TEXT S1 [file mBio.01846-20-s0001.docx]

**Supplementary text**

***Prochlorococcus* rely on microbial interactions rather than on chlorotic resting stages to survive long-term nutrient starvation**

Dalit Roth-Rosenberg^a*^, Dikla Aharonovich^a*^, Tal Luzzatto-Knaan^a*^, Angela Vogts^b^, Luca Zoccarato^c^, Falk Eigemann^b^, Noam Nago^a^, Hans-Peter Grossart^c,d^, Maren Voss^b^ and Daniel Sher #^a^

^a^ Department of Marine Biology, Leon H. Charney School of Marine Sciences, University of Haifa, 199 Aba Khoushy Ave. Mount Carmel, Haifa 3498838, Israel.

^b^ Leibniz-Institute for Baltic Sea Research, Seestrasse 15, D-18119 Warnemuende, Germany.

^c^ Department of Experimental Limnology, Leibniz-Institute of Freshwater Ecology and Inland Fisheries, Alte Fischerhuette 2, D-16775 Stechlin, Germany.

^d^ Potsdam University, Institute of Biochemistry and Biology, Maulbeeralle 2, D-14469 Potsdam, Germany.

**Can mis-sorted cells explain the presence of non-active cells in the high-fl population, and of active cells in the low- and mid-fl populations?**

FACS-sorting high-, mid- and low-fl populations from *Prochlorococcus* cultures resulted in samples that are highly enriched in the sorted populations. However, the sorted populations are not completely pure, with some incorrectly sorted cells observed when the sorted populations were re-analyzed by flow cytometry (e.g. mid- or low-fl cells in the sorted high-fl population, Table S1A). These incorrectly sorted cells could affect the interpretation of the single-cell uptake rates, for example, the presence of inactive cells in the high-fl population could be interpreted either as a real biological phenomenon (cells with high autofluorescence that are nevertheless inactive) or as the result of incorrectly sorted cells belonging to the mid- or low-fl populations (assuming the mid- and low-fl cells are inactive). To test this hypothesis, we first determined, for each cell in the NanoSIMS analysis, whether it was active or inactive, defining inactive cells as those with C and N uptake rates in the range of the control, i.e. glutaraldehyde killed cells (Table S1b, Killed cells C_max_ = 0.765 fg cell^-1^ day^-1^ and N_max_ = 0.215 fg cell^-1^ day^-1^. We then compared the observed number of active cells to the expected number of active cells, based on the number of high-, mid- and low-fl cells after FACS sorting, and assuming only the high-fl cells are active. For the datasets shown in Fig. 2 and Fig. S2B, the hypothesis that the number of active cells could be explained by the number of high-fl cells after sorting was rejected (X^2^ test, DF=2, p<0.01, X^2^=24.4 and n=236 cells for Fig 2, X^2^=336.1 and n=415 cells for Fig. S2). There were fewer active cells in the high-fl populations than expected (68% and 64% active cells Table S1b, compared to 94% and 84.5% high-fl cells, respectively, in the high-fl subpopulation Table S1A), suggesting that some high-fl cells are inactive. Conversely, there were more active cells than expected in the mid- and low-fl population (Table S1), suggesting that some cells in these populations can be active.
